# Comparative analysis of the mammalian *WNT4 *promoter

**DOI:** 10.1186/1471-2164-10-416

**Published:** 2009-09-06

**Authors:** Hongshi Yu, Andrew J Pask, Geoffrey Shaw, Marilyn B Renfree

**Affiliations:** 1 ARC Centre of Excellence in Kangaroo Genomics, Department of Zoology, The University of Melbourne, Victoria 3010, Australia

## Abstract

**Background:**

WNT4 is a critical signalling molecule in embryogenesis and homeostasis, but the elements that control its transcriptional regulation are largely unknown. This study uses comparative cross species sequence and functional analyses between humans and a marsupial (the tammar wallaby,*Macropus eugenii*) to refine the mammalian *Wnt4 *promoter.

**Results:**

We have defined a highly conserved 89 bp minimal promoter region in human *WNT4 *by comparative analysis with the tammar wallaby. There are many conserved transcription factor binding sites in the proximal promoter region, including SP1, MyoD, NFκB and AP2, as well as highly conserved CpG islands within the human, mouse and marsupial promoters, suggesting that DNA methylation may play an important role in *WNT4 *transcriptional regulation.

**Conclusion:**

Using a marsupial model, we have been able to provide new information on the transcriptional regulators in the promoter of this essential mammalian developmental gene, *WNT4*. These transcription factor binding sites and CpG islands are highly conserved in two disparate mammals, and are likely key controlling elements in the regulation of this essential developmental gene.

## Background

Wingless-type MMTV integration site family, member 4 (WNT4) is a locally acting signalling molecule, regulating cell-cell interactions, proliferation, differentiation, migration, and gene activation [1-10]. It is a highly conserved gene within mammals and has multiple roles in organogenesis and homeostasis and is of special interest in the sexual differentiation of the ovary [11-24]. While it can elicit its effects through the "canonical" WNT/β-catenin signalling pathway [5], it mainly acts through "non-canonical" signalling pathways. The WNT4 signalling pathway is mediated by the factor c-Jun N-terminal Kinase (JNK) in both the frog eye and human kidney development [16,25,26]. While WNT4 function is well defined, the underlying mechanisms that regulate its expression are still largely unknown.

Marsupials are mammals that diverged from the eutherian mammal lineage around 130 million years ago. Marsupials differ from other mammals in subtle ways especially in their mode of reproduction [27]. We use the tammar wallaby as the model marsupial because most sexual differentiation occurs after birth, unlike in eutherian mammals where differentiation of the testis and ovary occurs *in utero *[28,29]. We now know that *WNT4 *appears to be as important in differentiation of the marsupial ovary as it is in eutherians [13], suggesting that this gene has been highly conserved during therian (eutherian and marsupial) evolution. Despite its importance as a developmental regulator, the regulation of *WNT4 *has not been clearly defined. It is hard to distinguish functional from non-functional sequences in the promoter region by comparing closely related species such as mouse and human. It was therefore of interest to examine the promoter region of this important gene in representatives of the two therian mammal groups that diverged over 130 million years ago.

Initiation of transcription is the primary mechanism by which genes are regulated and is dependent on the structure of the promoter region and the corresponding transcription factor binding sites. There has been some analysis of the *WNT4 *promoter region. The human *WNT4 *immediate 5'-flanking region (1.2 kb) has promoter activity, but the critical promoter elements have not yet been defined [30]. Although WT1 down-regulates *WNT4 *gene expression *in vitro*, no active WT1 binding sites have been identified in the 1.2 kb promoter region by reporter analysis [30]. p21 is a negative transcriptional regulator of *Wnt4 *expression in keratinocyte growth control [31]. Furthermore, P21 binding to the *Wnt4 *promoter is mediated through the transcriptional factor E2F-1 [32], and there is an E2F-1 binding site near the TATA box-proximal region of the mouse *Wnt4 *promoter [31]. In gonadal development, fibroblast growth factor 9 (FGF9) and WNT4 appear to act as antagonistic signals to regulate mammalian sexual differentiation [33]. In mouse kidney development, the transcriptional factor PAX2 activates *Wnt4 *expression *in vitro *and *in vivo*, and also directly activates human *WNT4 *promoter activity [26]. Furthermore, there are three PAX2 recognition motifs in the 5'-flanking sequence of the human *WNT4 *gene [26] but the precise pathway of transcriptional regulation of *WNT4 *gene in renal development is still unclear. There is a novel response element (RRRCWWGYYY) in the human *WNT4 *promoter that confers p63- and p73-specific activation [34]. Together, these studies contribute to our understanding of *WNT4 *transcriptional regulation mechanisms, but how transcriptional modulation and chromatin structure remodelling are controlled by the promoter is still poorly understood and as yet there is no complete analysis of the *WNT4 *promoter.

Comparative genomics is a powerful way to identify conserved elements of genes and promoter regions likely to be essential for their function [35]. Selecting suitable species is the key for evolutionary comparisons. If closely related species are selected, it is very hard to distinguish the random sequences from the functional sequences. Marsupials provide an ideal model for the identification of conserved mammalian gene features since they diverged from mice and humans over 130 million years ago [36]. Elements conserved between mice and humans (eutherian mammals) and the tammar wallaby (a marsupial mammal) over this long period of divergent evolution are therefore likely to be critical to their function.

This is the first study of the *WNT4 *promoter in any non-eutherian mammal. We used comparative genomics to isolate conserved mammalian promoter elements, and examined their function in promoter construct assays in both tammar primary cell lines as well as human cell lines. This has enabled identification of the conserved elements of the promoter and transcriptional factor binding sites (TFBS) likely to be essential in the regulation of *WNT4*. We have also analysed the human *WNT4 *promoter in these cell lines and compared their activities to further define the function of this region.

## Results

### Cloning of tammar and human *WNT4 *5' flanking sequence

Since there were no EcoRI restriction enzyme sites identified in the tammar wallaby *WNT4 *cDNA sequence, this enzyme was used to create a shotgun library of a *WNT4 *containing tammar BAC clone (166C18 from *Macropus eugenii *BAC library; ME_KBa, Arizona Genomics Institute, USA) [13] in pBluescript II SK (-) vector. Resulting clones were PCR screened for the presence of exon 1. One clone was identified with a 6.5 kb insert and sequence analysis confirmed that it contained about 3 kb upstream of the tammar *WNT4 *gene. The transcription start site was determined according to our previous analysis of the tammar *WNT4 *gene [13] and all of transcripts/isoforms were obtained with the same untranslated region (data not shown). The human *WNT4 *5' upstream sequence was retrieved from the Ensembl database . Primers (see Table 1) were designed to amplify the orthologous 3 kb DNA fragment and smaller regions from human genomic DNA. Primers (see Table 1) were engineered to contain either Acc65I or XhoI restriction sites to enable cloning into the pGL4.10 reporter construct. A high GC content PCR kit was used to obtain the fragments due to the abnormally high GC content of these regions (82% GC content in tammar 299 bp and 600 bp constructs).

**Table 1:** Primers designed for promoter analysis

***Primers***	***Sequence*(5'→ 3')**	***function***
p hWNT4F1	AAGGTACCGACAGGACAGAGGCAGAGGT	with hRL, 1968 bp
p hWNT4F2	AAGGTACCCACCATCCCAGCACCCAA	with hRL, 1608 bp
p hWNT4F3	AAGGTACCGGAAGGCTGTAGGGAGGTG	with hRL, 895 bp
p hWNT4F4	AAGGTACCCGGCGCTGACAGTCTGGT	with hRL, 255 bp
p hWNT4RL	ATCTCGAGGAAGACGGCGAAGACGAG	reverse primer
p hWNT4 FD	AAGGTACCCAGGACAGAGGCAGAGGTAGG	with hRD, 1061 bp
p hWNT4 RD	ATCTCGAGTTGGGCAGCTTGGGACAG	reverse primer
p tWNT4F1	GAGGTACCGAAGTAACTCAAAAAGGA	with tRL, 2839 bp
p tWNT4F2	ACGGTACCCAGAGCAATCAGGGAAGG	with tRL, 1998 bp
p tWNT4F3	ACGGTACCCCCAGAGCGAATAAGTGA	with tRL, 994 bp
p tWNT4F4	ACGGTACCTGGCCAGCTCCGAGG	with tRL, 616 bp
p tWNT4F5	ACGGTACCCAGCGCAGAGACCAGGGA	with tRL, 315 bp
p tWNT4RL	AACTCGAGCGAGAAGACGGCGAAGAC	reverse primer
p tWNT4 FD	ATGGTACCGCACCTACTATGTCCCAAGC	with tRD, 2168 bp
p tWNT4 RD	TTCTCGAGCTTCCCCTTCCTGCTCCC	reverse primer
p hWNT4F	TGAGTCAAAGCGTGTAAAGC	Human total
p hWNT4R	CTGAGAAGACGGCGAAGA	promoter regions
E1F	CGGGCGGCACGATGAGT	Screening
E1R	CCAGTTGCTGGCGGCTG	exon1
tWNT4F	GAAACCGACGGTGGAAC	Checking
tWNT4R	AGGAGATGGCATAGACGAA	tWNT4 expression (102 bp)
hWNT4F	TCTGACAACATCGCCTACGG	Checking
hWNT4R	GGCACTTGCATTCCACCC	hWNT4 expression (160 bp)

p: promoter; h: human; t: tammar; F: forward primer; R: reverse primer; D: deletion

### Comparative analysis of the *WNT4 *upstream and downstream genomic sequences

**Figure 1:**
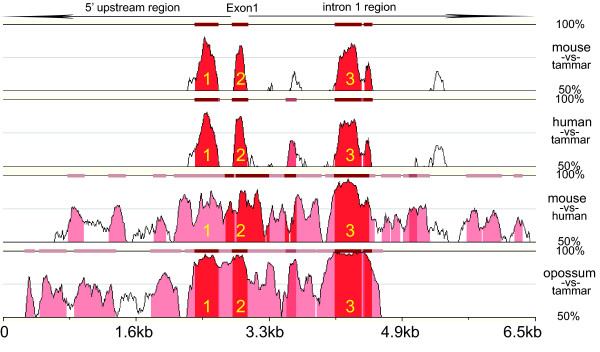
**Comparison of *WNT4 *exon1 upstream and downstream among tammar, opossum, human and mouse**. Cross species genomic analysis shows that the highly conserved regions occur within *WNT4 *exon 1 [labelled 2], the proximal promoter region [labelled 1] and downstream regulatory motifs [labelled 3] by the MULAN program. The red blocks represent more than 75% identity (Sequences details are in the complementary part). The genomic sequences were retrieved from UCSC Genome Browser : human (chr1: 22,338,357-22,344,889, released in Mar 2006); mouse (chr4: 136,830,708-136,837,240, release in July 2007); opossum (scaffold_18713: 294,346-298,919, released in Oct 2004).

Sequences from the tammar, opossum (representing another marsupial species; *Monodelphis domestica*), human and mouse were aligned using MULAN  to investigate the conservation of the *WNT4 *promoter region. Analysis was performed over an approximately 6 kb region from each species, consisting of 3 kb of upstream and 3 kb of downstream of *WNT4 *exon 1. Due to gaps in the genome assembly, only 1.6 kb of sequence was available for the opossum downstream of exon 1. The analysis identified three clearly conserved regions, one covering exon 1 as predicted, but another was found in the immediate 5' region and one other in intron 1 (Figure 1 and Additional file 1).

### Identification of the conserved *WNT4 *promoter region

**Figure 2:**
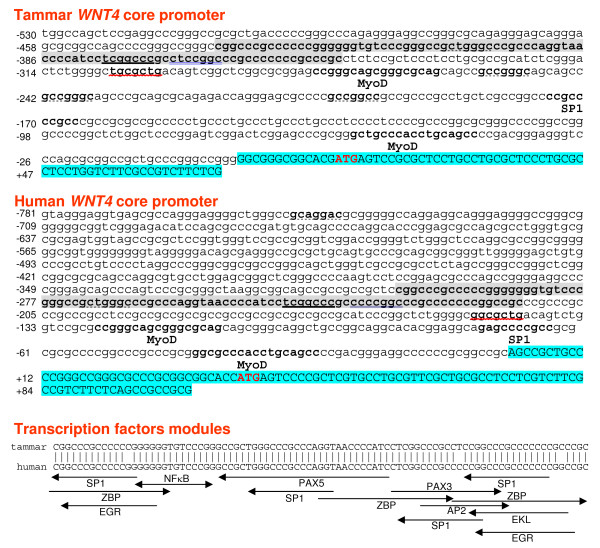
**Comparative analyses of *WNT4 *proximal promoter in tammar and human**. Tammar and human *WNT4 *proximal promoter sequences and common potential transcription factors including TF modules are identified. They share the same minimal promoter characteristics, indicating similar mechanisms initiate *WNT4 *transcription and regulate its expression in both species. The potential putative transcription factors were labelled under their corresponding sequence. The red "ATG" indicated the start codon. The left arrow represents the DNA strand (-) of transcription factor binding site. The right arrow stands for the DNA strand (+) of transcription factor binding site. Core-search identified 4 core motifs located within the promoter region. **TCGGCCC **(black line) represents highly conserved motif 1; CTCCGGC (double blue line) stands for motif 2; **GCTGGCC **(dotted line) is motif 3;**TGCGCTG **(red line) is motif 4.

To further explore the conservation of the human and tammar *WNT4 *promoter, we compared minimal promoter sequences (defined with scanning deletion analyses of *WNT4 *promoters below) with the GenoMatix program DiAlign TF (Multiple Alignment plus Transcription Factor Sites) . We identified a highly conserved transcriptional module (89 bp in length, and sharing 97% homology between human and tammar (Figure 2 grey shading) that contains multiple TFBS including ZBP (Zinc binding protein factors), SP1 (Stimulating protein 1), EGR (early growth response), NFκB (Nuclear factor kappa B/c-rel), PAX5 (PAX-5 B-cell-specific activator protein), PAX3 (PAX-3 binding sites), AP2 (Activator protein 2) and EKLF (Basic and erythroid krueppel like factors) (Figure 2). There are also three other highly conserved TFBS, outside of this region, two for MyoD (Myoblast determining factors) and one for SP1. We also investigated the conservation of motifs in the distal 5' flanking regions from tammar, human and mouse (approximately -2874 bp in each). We have identified four conserved motifs (shown with different type underlines in Figure 2); motif 1 (TCGGCCC recognized by the vertebrate matrix family SP1 and ZBP), motif 2 (CTCCGGC recognized by the vertebrate matrix family AP2, EGR, PAX3, SP1 and ZBP), motif 3 (GCTGGCC) and motif 4 (GGCGCTG recognized by the vertebrate matrix family EGR, SP1 and ZBP).

**Figure 3:**
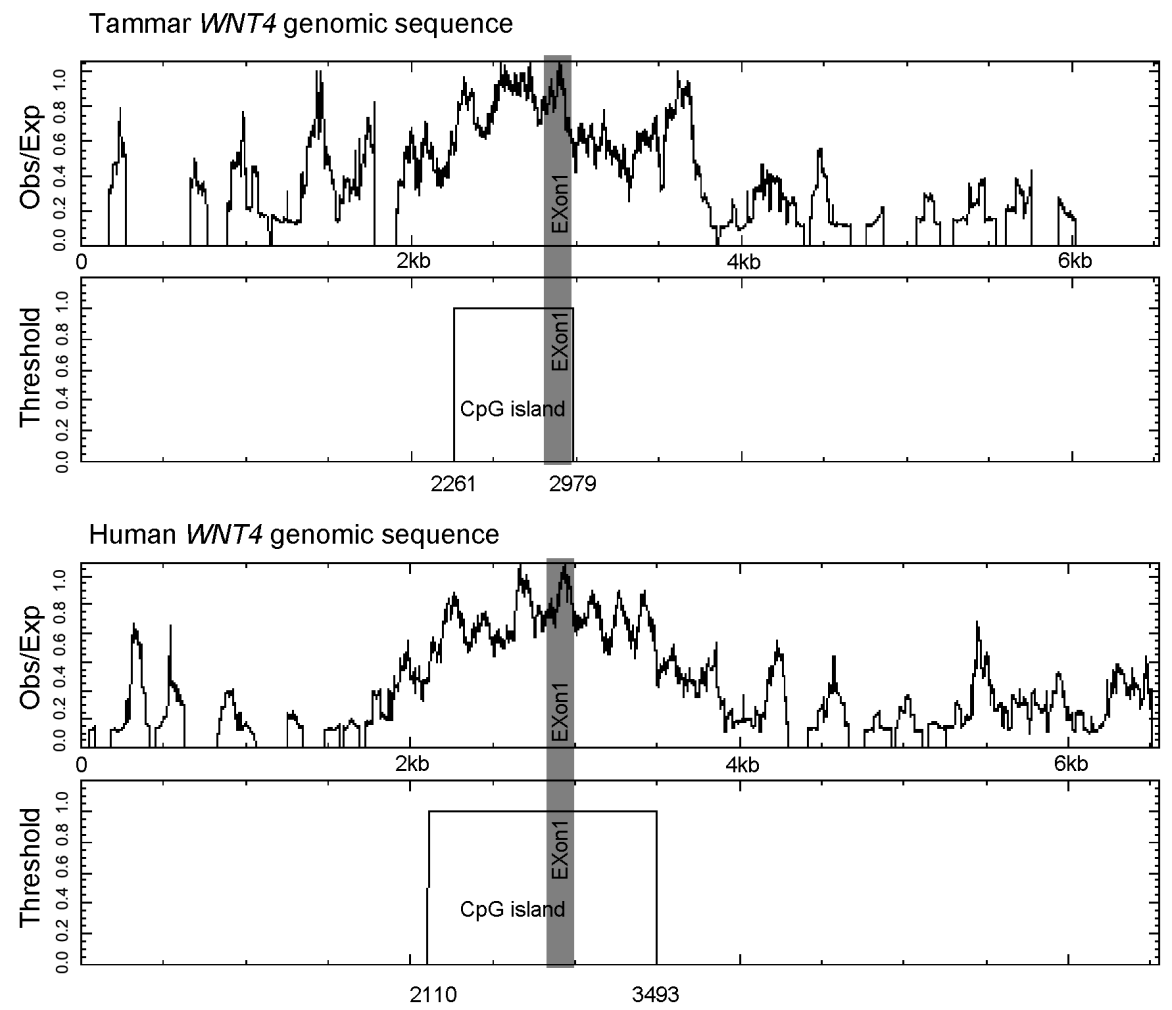
**CpG islands analysis of *WNT4 *promoter regions**. High GC content exists in the promoter regions of both tammar and human. CpG islands in the tammar begin at the proximal 5' flanking region and end at exon 1. CpG islands in the human promoter region occur at the proximal 5' flanking region and extend into intron 1-2. Observed/Expected ratio >0.60; the length of CpG islands >200.

We did not identify a TATA box immediately 5' of the transcription start site and no CCAAT sequence was identified in the proximal promoter region. However, very high GC content and CpG islands are conserved features of this region (Figure 3).

### Functional analysis of the *Wnt4 *promoter region

**Figure 4:**
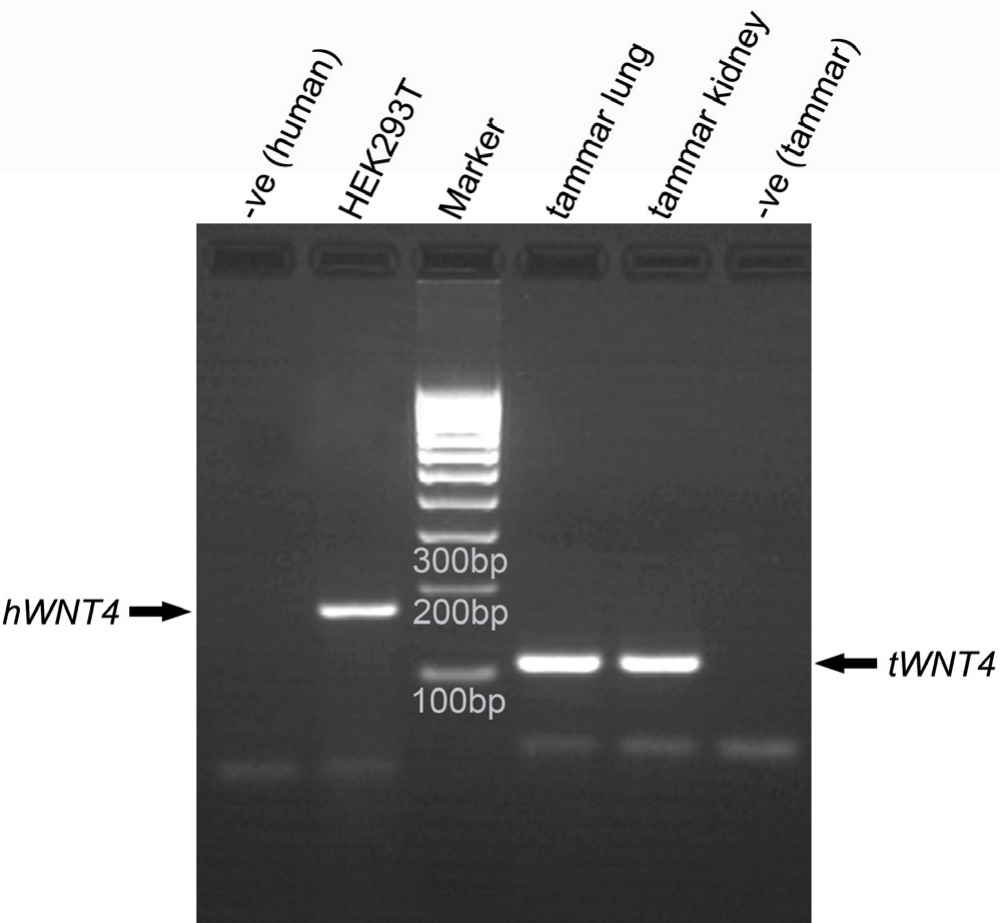
***WNT4 *expressions in different cell types**. Human *WNT4 *gene expression (indicated by a 160 bp RT-PCR product) was seen in human embryonic kidney HEK293T cells; both tammar primary lung cells and kidney cells express the tammar *WNT4 *gene (indicated by a 102 bp RT-PCR product). Gel lanes are marked as follows: M, Marker; 1,5, Negative Control; 2, HEK293T cDNA; 3, tammar lung cell cDNA; 4, tammar kidney cell cDNA.

Before examination of promoter function, *WNT4 *expression was confirmed in the primary cell lines. The primary cell lines used for analyses were derived from neonatal tammar wallaby lung and kidney, and an established human embryonic kidney cell line (HEK293T). Primers (see Table 1) spanning introns confirmed the expression of *WNT4 *in all cell lines (Figure 4).

**Figure 5:**
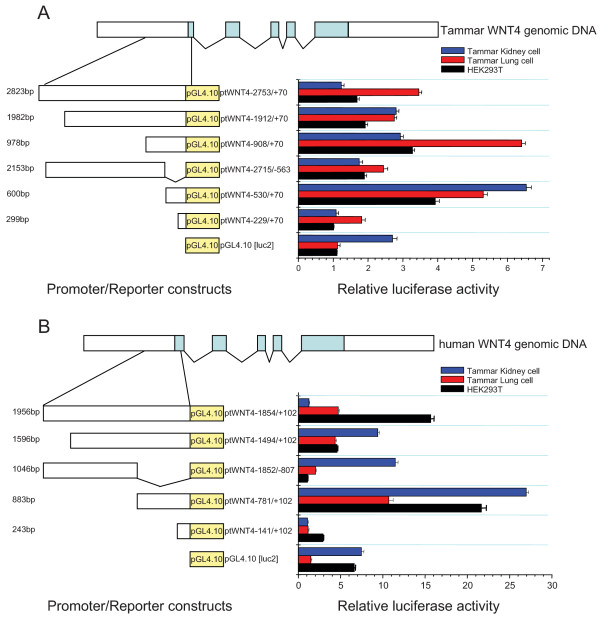
**Scanning deletion analyses *WNT4 *promoters in different cell lines**. The top panel illustrates the tammar/human *WNT4 *gene structure. The left side of the diagram illustrates the constructs consisting of the different promoter regions fused upstream of a luciferase reporter, and the right illustrates the relative luciferase activity derived from the constructs using tammar kidney cells (blue), tammar lung cells (red) and human HEK293T cells (black). (A) The -530/+70 fragment in tammar *WNT4 *proximal promoter showed the maximum activity in the tammar kidney cell line and HEK293T cell line while the -980/+70 fragment showed the maximum activity in the tammar lung cell line; (B) the -781/+102 fragment displayed the maximum activity in all of three cell lines.

Deletion analysis of the putative promoter was used to uncover the regions necessary for the transcriptional activity in kidney and lung cell lines. 5' upstream fragments were fused to a luciferase reporter vector (PGL4.10, Promega, USA). The promoter-luc2 constructs and basic vector pGL4.10 were then used to transfect three different cell lines, human HEK293T cells and tammar lung and kidney cells (Figure 5). Fragments are described in bp either side of the start codon.

Maximum promoter activity was found in the tammar -530/+70 fragment within tammar kidney primary cell lines and human HEK293T kidney cell lines. Smaller constructs (-229/+70) showed very low promoter activity in the above two cell lines. Larger fragments (up to -2753), had reduced activity, indicative of transcriptional repressors present further 5' in the promoter region. Transcriptional inhibitor binding sites appeared to be present in the -2753/-908 regions of the tammar promoter because the transcriptional activity was lower in these longer regions in all three different cell lines and much lower than in the -530/+70 (kidney) and -980/+70 (lung) regions. However, in the tammar lung primary cell line, the -980/+70 showed the highest promoter activity, above that of the -530/+70 fragment from kidney. This suggests that important lung cell TFBS are likely to be located within this region (-530 to - 980) to increase gene function by protein-DNA or protein-protein interactions. Noticeably, we also found that the fragment -229/+70 has almost double the activity compared to "no insert" control in the tammar lung primary cell lines, suggesting the different cellular context is very important for activation of gene transcription. In addition, minimal promoter refers to the shortest fragment that can drive the gene initiation or expression. Taken together, these data suggest that the tammar *WNT4 *minimal promoter region lies in the -530/+70 region *in vitro*.

These findings are consistent with previous analyses, which suggest that the human *WNT4 *promoter is located in the -781/+102 fragment and larger fragments (up to -1854 bp) showed reduced promoter activity suggesting negative regulator binding sites within this region [30]. Furthermore, the immediate 5' region (-141 bp) also had no promoter activity. Interestingly, we also found that human promoter constructs had higher relative expression in tammar cell lines than the corresponding tammar promoter constructs. This may be due to subtle differences in the regions covered by fragments derived from each species for analysis. However, while the activities of promoters were variable between species, both marsupial and human promoter regions show similar trends between comparable promoter regions.

### Chromosome mapping

**Figure 6:**
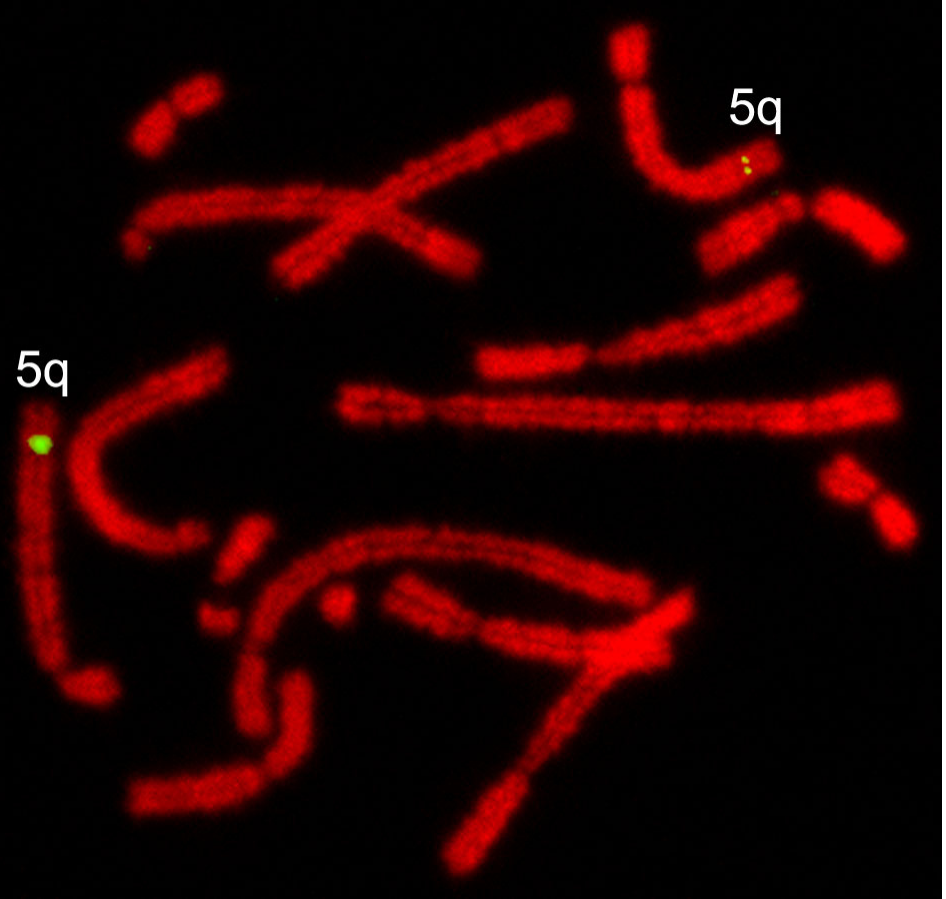
**Fluorescence in situ hybridization of the tammar *WNT4 *to tammar metaphase chromosome spreads**. Tammar *WNT4 *gene was localized at the long arm of chromosome 5, consistent with WNT4 autosomal location in other species.

The chromosomal location of tammar *WNT4 *gene was determined by fluorescent *in situ *hybridization to metaphase plates. *WNT4 *is autosomal, as in all other mammals (Table 2) and is on the long arm of tammar chromosome 5 (Figure 6). Primate *WNT4*, including human [37,38], chimp and macaque is on Chromosome 1, whereas *WNT4 *of cow and dog is on chromosome 2.

**Table 2:** Chromosome localization of *WNT4 *gene in known species

***species***	***Chromosome Localization ***	***species***	***Chromosome Localization ***
human	Chr1, p36.12	tammar	Chr5, q
chimp	Chr1, 22.3	platypus	Chr5, q
macaque	Chr1, 24.7	chicken	Chr21, 6.51
cow	Chr2, 124.46	zebrafish	Chr9, 30.32
dog	Chr2, 80.01	Medaka	Chr5, 24.19
mouse	Chr4, D3	Tetraodon	Chr11, 10.4
rat	Chr5, 156.1	stickleback	groupXVII, 11.16
opossum	Chr4, 356.08		

*Note*: The chromosome localizations of *WNT4 *gene in chimp, macaque, cow, dog, mouse, rat, opossum, chicken, zebrafish, Medaka, Tetraodon and stickleback were assigned according to homepage of Ensembl  or NCBI , and platypus *WNT4 *was localized by Grafodatskaya et al [53].

## Discussion

*WNT4 *is an essential gene in embryogenesis, critical for the development of kidney, adrenal, gonad, mammary gland and eye [10,16,22,23,39]. Despite its many functions, the transcriptional regulation of the *WNT4 *gene remains poorly characterised. In this comparative analysis of the mammalian *WNT4 *promoter, we have refined the promoter and identified evolutionarily conserved TFBS that appear to be important for *WNT4 *transcriptional regulation in kidney and lung, in all mammals including man. Since these regions have been conserved in sequence and function for over 130 million years [36], they are likely to represent promoter and enhancer regions critical for mammalian *WNT4 *gene regulation.

We found several conserved putative TFBS in both the human and tammar promoter regions. These were mainly conserved within an 89 bp transcription module, designated as TF module I, which shared a 97% identity between human and tammar (Figure 2). We also identified a conserved MyoD-SP1-MyoD transcription module between module I and the transcription start site (TSS), which could have a central role in transcriptional regulation. SP1 is one of the first eukaryotic transcription factors to stimulate or repress transcription by binding/competing to the GC/GT elements or recruiting chromatin remodelers [40,41]. Except for the 4 common SP1 binding sites shared across species, the tammar has an additional 9 and human an additional 7 SP1 binding sites predicted between the TSS and TF module I (data not shown). SP1 therefore could have an essential role in initiation of *WNT4 *gene expression. We found a conserved binding site for the Zinc finger binding protein (ZBP), a transcriptional regulator involved in cell proliferation and differentiation [42,43]. A conserved Nuclear Factor κB (NFκB) binding site was also found in TF module I. NFκB regulates target genes at several levels including controlled cytoplasmic-nuclear shuttling and modulation of transcriptional activity [44]. We also identified PAX5 and PAX3 TFBS in the TF Module I. Interestingly, PAX2 has been shown to bind to the *WNT4 *promoter and activate its expression during the kidney development [26]. However, this motif does not appear to be conserved in the tammar core promoter. The sequence about 0.45 kb upstream of the transcriptional start site in the human promoter had homology to both the consensus PAX2 motif proposed by Brophy *et al*., [45] and to the PAX5 motif predicated by MatInspector software [26]. There is some crossover between PAX2 and PAX5 when binding the mb-1 probe and recruitment of Est-1 [46]. Taken together, this motif is highly conserved and may act as a PAX2 binding site depending on the cellular environment. MyoD, another identified transcription factor binding to this region, directly regulates gene expression by modulation of the chromatin structure in myogenesis [47]. *WNT4 *is expressed in tammar and human muscle and involved in the control of smooth muscle cell fate [3,13] but the factors that regulate its expression in this process are unknown. Our findings suggest that MyoD could play a conserved role in the regulation of *WNT4 *gene expression.

Both human and tammar proximal promoters lack the typical canonical TATA box and CCAAT box. Instead, they feature a high GC content and CpG islands, suggesting other mechanisms, such as DNA methylation and chromatin remodelling are required to initiate *WNT4 *transcription. High GC content and CpG islands are typical signatures of promoters that lack a TATA box and the usual initiation elements [48]. DNA methylation can occur on the cytosine base of high GC content regions, and inhibit transcription through the binding of methyl-CpG-binding proteins or by blocking binding of specific transcriptional activators [49,50]. Thus, DNA methylation and demethylation can act as an alternative mechanism to regulate gene activation. Most housekeeping genes are expressed constitutively where CpG islands are unmethylated, whereas the *WNT4 *gene is a spatially and temporally regulated gene during development. Our analysis suggests the regulation of *WNT4 *is mediated, at least in part, by DNA methylation. However, to confirm this method of *WNT4 *regulatory control, it would be essential to investigate the DNA methylation status of the GC rich region in different tissues. Interestingly, there is a TATA box in the mouse proximal promoter, inferring that the mouse may have developed an additional regulatory region. However, the mouse *Wnt4 *promoter still retains the proximal GC rich region as seen in humans and tammar wallaby.

We also identified a highly conserved region in intron 1 of the tammar, opossum, human and mouse, which could contain downstream regulatory or enhancer elements. However, when blasting these highly conserved sequences against EST databases in GenBank, we found that they are expressed in the fetal heart of humans from 19 weeks and also in the Wolffian ducts of embryonic day 12 mice. Thus, this region may represent a new gene which overlaps with *WNT4 *or a rare alternative transcript. Nevertheless, further investigations are needed to define its expression and function, and to determine its relationship, if any, to *WNT4*.

In order to compare the conserved promoter activity of the tammar and human *WNT4 *genes, we performed cross-transfection of promoter constructs in both human and tammar cell lines. This is the first functional analysis of a tammar wallaby gene promoter, confirming that primary cell lines can be useful for this type of analysis. The human constructs had a higher relative expression in tammar cell lines than the tammar constructs. One possible reason is that there are more TFBS in human constructs to activate gene expression or fewer inhibitors to repress gene expression. However, similar results were obtained for orthologous promoter regions in both species across both cell lines. This shows that the TFBS have maintained conserved functions over at least 130 million years, indicating they are likely to be critical for gene function. Our deletion analysis further refined which parts of the conserved region contain the functional promoter region of the *WNT4 *gene and the putative TFBS required for its activation. Although this study has identified numerous highly conserved TFBSs, further study, including ChIP/EMSA and mutation and transfection analysis is needed to define the precise role of each transcription factor in the regulation of the mammalian *WNT4 *gene.

## Conclusion

This is the first comparative study of the mammalian *WNT4 *promoter. Using a marsupial model, we have been able to provide new information on the transcriptional regulators in the promoter of this essential mammalian developmental gene. These transcription factor binding sites and CpG islands are highly conserved in two disparate mammals, and are likely key controlling elements in the regulation of this essential developmental gene. These findings provide a platform for further targeted approaches to defining the precise function of each TFBS in *WNT4 *regulation.

## Methods

### Cloning the 5' Upstream Region of *WNT4 *Gene in Tammar and Human

The transcription start site was determined according to our previous analysis of tammar *WNT4 *gene [13]. Briefly, we amplified tammar *WNT4 *partial sequence with degenerated primers, and then employed 5'RACE and 3'RACE to obtain the upstream and downstream sequences from this partial sequence. We just got one band with 5'RACE and multiple bands with 3'RACE, and confirmed these transcripts/isoforms with primers from 5'UTR and 3'UTR. The tammar BAC library was screened according to previous methods with the tammar ^32^P^-^labelled *WNT4 *partial sequence as a probe [13]. BAC DNA was digested with EcoRI, and shotgun subcloned into the pBluescript II SK (-) vector. Primers within tammar exon 1 (E1F and E1R, Table 1), were used to screen the clones. An equivalent region was amplified (primers: phWNT4F and phWNT4R, Table 1) from the human genomic based on the human WNT4 genomic sequence .

### In Silico Analysis of the mammalian *WNT4 *Regulatory Region

Human, mouse and opossum *WNT4 *genomic sequences including 3 kb upstream and downstream of the start codon were retrieved from the Ensembl website . Sequence alignments were performed using MULAN .

The potential TFBS of the minimal promoter region defined by deletion analysis were predicated by AliBaba2.1  and MULAN . Core-search to define the unknown motifs in the 5' flanking regions (2874 bp) of *WNT4 *gene in the tammar (GenBank: EU003446), mouse and human was carried out using GEMS, Genomatix software . CpG islands were predicated by cpgplot .

### Reverse Transcriptase Polymerase Chain Reaction (RT-PCR)

Intron spanning primers were used to confirm endogenous *WNT4 *gene expression in the cell lines chosen for transfections. Tammar *WNT4 *was amplified with tWNT4F and tWNT4R (Table 1) [13] while human was amplified using hWNT4F and hWNT4R (Table 1). Total RNA was isolated from cells using the GeneElute kit (Sigma, Castle Hill, NSW, Australia), and the quality and quantity of total RNA verified with the NanoDrop^® ^ND-1000 spectrophotometer (NanoDrop Technologies, Wilmington, USA). 2 μg of total RNA was DNase-treated with DNA-free (Ambion Inc., Austin, Texas, USA). 1 μg of total RNA was reverse-transcribed using SuperScript III (Invitrogen, California, USA). PCR was performed in a 50 μl reaction containing 10 mM Tris-HCl, pH 8.3, 1.5 mM MgCl_2_, 50 mM KCl, 200 μM dNTP, 0.2 μM each primer, 1 U Taq DNA polymerase (Promega, Wisconsin, USA). Amplification conditions were: 94°C, 30 s; 56°, 30 s, 72°C, 30 s for 38 cycles.

### Generation of Luciferase Reporter Constructs

Primers were engineered with Acc65I and Xhol restricted enzyme sites to allow directional cloning of fragments into a luciferase expression vector (pGL4.10, Promega, USA). PCR was performed (primer sequences, Table 1) with GC-RICH PCR System (Roche, Germany) according to the manufacturer's protocol because of high GC content in the promoter region. The PCR products and vector were then digested with these two enzymes and ligated to screen the promoter-luc2 constructs. All PCR products and constructs were purified with Qiagen gel extraction and purification kits (Qiagen, Doncaster, VIC, Australia), and the concentration of constructs determined with a NanoDrop N-1000 spectrophotometer (NanoDrop Technologies, Wilmington, USA).

### Cell Culture, Transient Transfection and Luciferase Assays

All cells were cultured at 37°C with 6% CO_2 _in a humidified incubator. Human embryonic kidney HEK293T cells were grown in Dulbecco's modified Eagle medium (DMEM) (GIBCO, Invitrogen) with 10% fetal bovine serum (FBS). Tammar lung primary cells (low passages) from Day 70 after birth removed pouch young (RPY) were grown in 50% DMEM containing 10% FBS and 50% AminoMax (GIBCO, Invitrogen) containing 15% fetal calf serum (FCS). Tammar kidney primary cells (low passages) from D30 after birth RPY were grown in 100% AminoMax with 15% FCS.

For transient transfection studies, cells were seeded into 24-well tissues culture plates for HEK293T or 12-well tissue plates for tammar primary cells at optimal densities. Approximately 24 hours after plating, HEK293T cells with 60-70% confluence or tammar primary cells with 80-90% confluence were transfected with human or tammar truncated promoter region fragment-luciferase reporter gene constructs and basal pGL4.10 luc2 vector (Promega, USA). Transfections mediated by Lipofectamine™ 2000 for HEK293T cells or Lipofectamine™ LTX Reagent with PLUS™ Reagent (Invitrogen, California, USA) for tammar primary cells were carried out according to the manufacturer's protocol using the same molar constructs in triplicate. Luciferase enzyme was extracted at 24 hours for HEK293T cells or 48 hours for tammar cells post-transfection using luciferase assay kit from Promega. Luciferase activity was measured using single-tube luminometers with injectors based on standard method, normalized to background levels of luciferase activity derived from negative control cells. In each group, we set the minimal value as "1", and then calculated the relative values for others. The data shown represent mean values from triplicate reads (± S.E.M).

### Chromosome Preparations and Fluorescence *in Situ *Hybridisation

Chromosome preparations were made from peripheral blood according to standard methods with minor modifications [51]. Briefly, the cells were cultivated in DME supplemented with 10% FCS. The peripheral lymphocytes were stimulated with either phytohemagglutinin (Murex) or staphylococcal enterotoxin A (Sigma). After 70.5 h, the cultures were treated with Colcemid (0.05 mg/ml) for one hour. The cells were then harvested by centrifugation, resuspended in hypotonic solution (0.4% KCl) for 30 min at 37°C, and pre-fixed by adding and resuspending 1 ml of fixative (methanol:acetic acid; 3:1). Cells were then washed three times with fixative and kept at -20°C overnight. Chromosomes were spread on dry, acetone-cleaned slides by applying drops of cell suspension from a height of 15 cm to 20 cm. Slides were air-dried for 24 h, dehydrated, air-dried, and stored at -80°C.

Chromosome *in situ *suppression (CISS) hybridization was performed as previously described with minor modifications [52]. The BAC genomic clone was screened with tammar *WNT4 *cDNA probe [13], labelled with dUTP-digoxygenin (DIG) by nick translation at 14°C for about one hour and co-precipitated with tammar wallaby Cot-1 DNA prior to hybridization. The probe was hybridized to tammar metaphase chromosome spreads at 37°C overnight. Hybridization was detected using mouse anti-DIG-FITC antibody (Serva). After CISS hybridization, the chromosome preparations were stained with DAPI (4, 6-diamidino-2-phenylindole) to visualize the chromosomes. Images were taken using a Zeiss microscope, and then merged and edited.

## Authors' contributions

All authors participated in the design of the study and analyzed the results. HY performed all of the experiments and drafted the manuscript. AJP, MBR and GS modified the manuscript. All authors read and approved the final manuscript.

## Supplementary Material

Additional file 1 **The three blocks with high homology identified by MULAN program in the WNT4 promoter region in human, mouse, opossum and tammar. Block No. 1, 2, and 3 correspond to 1, 2 and 3 in Figure 1 respectively.** Asterisks represent the nucleic acids conserved in all species. Click here for file
