# Supplementary material for: Comparative analysis of the mammalian WNT4 promoter
Source: BMC Genomics. 2009 Sep 6;10:416. doi: 10.1186/1471-2164-10-416 (PMC2758904; doi:10.1186/1471-2164-10-416)
Supplement: Additional file 1 — The three blocks with high homology identified by MULAN program in the WNT4 promoter region in human, mouse, opossum and tammar. Block No. 1, 2, and 3 correspond to 1, 2 and 3 in Figure 1 respectively. Asterisks represent the nucleic acids conserved in all species. [file 1471-2164-10-416-S1.pdf]

BLOCK NO. 1

```
mouse    GGCGGGACCCAGCAGCTGGGTCGCCGCGCCTCTAGCTCGGCCCAACTGGGCGGCGTGCGGCCAAGCGTGCCTGCAGCAGCGGGGCTGAGGCTCCGAGCGCGTCCGGGCGCCGGGCCGGG
human    GGCGGCGGCCGGGCAGCTGGGTCGCCGCGCCTCTAGCCGGGCCGGGCTCGGCGGCGCGCAGCCAGGCGTGCCTGGAGCGG----GCTCGGGCCCCAAGTCCTCCGGAGCGCCCAGCCGGG
opossum  GAGGGAGGCCGGGT------------------------------------ACGGAG-----------------GGAGCAG-----------------------GGAGCGT---------
tammar   GAGGGAGGCCGGGC------------------------------------GCAGAG-----------------GGAGCAG-----------------------GGAGCGC---------
         *  ** * **  *                                   * * *                 * *** *                      * ***

mouse    GAGGCCCCCCAGGAGAAGCCCAGTCCCG-----------------------CGGGCTCAGGACCGCCCCCGTGGGGGGGGGTTGTCCCACGCCGCTGGACCCGCCCAGGTAACCCCATC
human    GAGG--CCCCGGGAGCAGCCCAGTCCCGCGGGCTAAGGCGGCAGCCGCCGCCGCTCC--GGCCCGCCCCCGGGGGGG------TGTCCCGGGCCGCTGGGCCCGCCCAGGTAACCCCATC
opossum  -----------------GCCCAACCCCG--------------------GGCCGGGCC--GGCCCGCCCCCCGGGGGGG-----TGTCCCGGGCCGCTGGGCCCGCCCAGGTAACCCCATC
tammar   -----------------GGCCAGCCCCG--------------------GGCCGGGCC--GGCCCGCCCCCCGGGGGG------TGTCCCGGGCCGCTGGGCCCGCCCAGGTAACCCCATC
                          * ***  ****                       **  *   **  ********  *****      ******  ********  *******************

mouse    CTCGGCCCGCCCCCGGCCCGCCCCGCCGGCCGCCCGCCCGCCT-----------------------CCGCCGCCACCGCCGCATCCCGG--CTCTGGGGCGGCGCTGACAGTCGGGTCC-
human    CTCGGCCCGCCCCCGGCCCGCCCCCCCGGCCGCCCGCCCGCCCGCCCGCCTCCGCCGCCGCCGCCGCCGCCGCCGCCGCATCCCGG--CTCTGGGGCGGCGCTGACAGTCTGGTCCG
opossum  CTTGGCCCGCCCCCGGCCCGCCCCCCCGCCCGCCTCCCGGCTC--------------------------CCTCCTGCGCCGCATCTCAGGACTCTGGGGCTGCGCTGACAGTCGGCTCGG
tammar   CTCGGCCCGCCTCCGGCCCGCCCCCCCGCCCGCCTCTCCGCTC--------------------------CCTCCTGCGCCGCATCTCGGGACTCTGGGGCTGCGCTGACAGTCGGCTCGG
         ** ******** *********** *** *****    * **                          ** **  ********* * *   ********* *********** * **

mouse    ------GCCGGGCAGCGGGCGCAGCAGCGGGCGGCGCGGC--CGGCTG
human    C-----GCCGGGCAGCGGGCGCAGCAGCGGGCAG-GCTGC--CGGCAG
opossum  CGCGGAGCCGGGCAGCGGGCGCAGCAGCCGCCGG-GCAGC--CCGCAG
tammar   CGCGGAGCCGGGCAGCGGGCGCAGCAGCCGCCGG-GCAGCAGCCGCCG
               ********************** * * * ** **  * ** *
```

BLOCK NO. 2

```
mouse    GTCCGCGCGGCGCCCACCTGCCGCCCCGACGGGAGCCT---------TGCGGCCGCTGCCCCGGGCTGGGCGC---GCACGGCACCATGAGCCCCCGTTCG-TGCCTGCGGTCCCTGCGA
human    GCCCGCGGGC-GCCCACCTGCAGCCCCGACGGGAGGCCCCCCGCGGCCGCAGCCGCTGCCCCGGGCCGGGCGCCCGCGGCGGCACCATGAGTCCCCGCTCG-TGCCTGCGTTCGCTGCGC
opossum  GCCCGCGGGCTGCCCACCTGCAGCCCCGACGGGAGGGTCCCAG----CACGGCCGCTG-CCCGGGCCGGGGGC---CGGCTGCACGATGAGTCCGCGCTCCCTGCCTGCGCTCCCTGCGC
tammar   GCCCGCGGGCTGCCCACCTGCAGCCCCGACGGGAGGGTCCCAG----CGCGGCCGCTG-CCCGGGCCGGGGGC---GGGCGGCACGATGAGTCCGCGCTCC-TGCCTGCGCTCCCTGCGC
         * ***** *  ********** *************                    * ******* ******* *** **       * **** ***** ** ** **  ******** ** *****

mouse    CTCCTCGTCTT-CGCCGTGTTCTC-GGCCGCCGCGAGCAATTGGCTGTAAGTCGGGCCGGGGA------------CCGGGCGGG
human    CTCCTCGTCTT-CGCCGTCTTCTC-AGCCGCCGCGAGCAACTGGCTGTAAGTCTGGCCGGGG-------------CCGAGCGGG
opossum  CTCCTGGTCTTTCGCTGTCTTCTCAGGCCGCCGCCAGCAACTGGCTGTAAGTGGGGCTGGGGGTGAGGAAAGTCTCTGAGCAGG
tammar   CTCCTGGTCTT-CGCCGTCTTCTC-GGCAGCCGCCAGCAACTGGCTGTAAGTGGGGCTGGGGGAGAGGAGAGCCGCCGAGCAGG
         ***** ***** *** ** *****  ** ***** ***** *********** ***  ****                 * * ** **
```

BLOCK NO. 3

```
mouse    AGCTCTGCAGCCTTCCACTGTGGGCAGCCAGTGGGGAGCCAAG-CTCCTGTATTGATCTGAAAAGCATGAGGTGGAAGGGGGGATAGCCAACAGTGTAG-CTGGGGTTTCCATGGAGAC
human    AGCTCTGCCACCATCCGCTGTGGGCAGCCAGTGGGGAGCCAAG-CTCCAATATTGATCTGAAAAGCATGGGGTGGAA-GGGGGATCGCCAACAGTGTAG-CTGGGGTTTCCATGGAGAC
opossum  AGTCCTGTTTTATTTCGCTGC------CCA---GGGAGCCTAGACCCCAATATTGATCCGAGGACTAAAGGG-------GGATATAGCCAACAGTGTAGCCTGGGGTTTCCATGGAGAC
tammar   AGTCCTGTTTTATTTCGCTGC------CCA---GGGAGCCTAGACCCCAATATTGATCCGAGGACTAAAGGG-------GGATATAACCAACAGTGTAGCCTGGGGTTTCCATGGAGAC
         **  ***      * * ***         ***   ******* ** * **  ******** **  *   *   **         ***  **  *********** ******************

mouse    AG-TGGGAAAGGAAAAA----TTCTCTCTGCATCAAGACCAAAGAACAAATATCTCCCATGAAATTGCCCTGAGTGTCCAATGGCTGTTGCTATGAGGTCATTGTGGCCAGCCAGGGC--
human    AG-GGAGAGAGGAAAAAA---TTCTCTCTGCATCAAGACCAAAGAACAAATATCTCCCATGAAATTGCCCCGAGTGTCCAATGGCTGTTGCTATGAGGTCATTGTGGCCAGCCAGAGCCC
opossum  CGCGGAGAGAGGAAAAAAATTTTCTCTCTGCATGAAGACTGGAGAACAAACGTCTCCCATGAGTTTGCCTTAAGTGTCCACTGGCTGTTGCTATGAGGTCATTGTGGCCTGCCGGG----
tammar   CGAGGAGAGAGGAAAAAAATTTTCTCTCTGCATGAAGACTGGAGAACAAACGTCTCCCATGAGTTTGTCTTCAGTGTCCACTGGCTGTTGCTATGAGGTCATTGTGGCCTGCCGGG----
          *  * ** ********    ************ *****     ********  ********** *** *    ******** ***************************** *** *

mouse    -CCTGGAGACTGGAAAGGAG-TGTGTTC-GTTGGGGTCAGAGCCACCCAGAGCTTGGGAGACTGAGGTGATGTGGAATTCTCCCAGCTGTTCCGAGAGCTCGC
human    TCCAG----GCTGGAAGGAG-TGTGTTC-GTTGGGGTCAGAGCCACCCAGGGCTTGGGGGACGGAGGTGATGTGGAATTCTCCCAGCTGTTCCGAGAGCAAAC
opossum  -CCTG----CTGGGAAGGAGTTGTGTTCAGTTGGGGTCAGAGCCACCGGGGAGTTTGGGGACTGAGGTGATGTGGAACTTTCCCAGCTGCTCCGGAGACCCAC
tammar   -CCTG----CTGGGAAGGAGCTGTGTTCAGTTGGGGTCAGAGCCACCGGGGAGTTTGGGGACTGAGGTGATGTGGAACTTTCCCAGCTGCTCAGGAGACCCAC
          ** *       * ****** ******* *****************  *    ** ** *** ************ * ********* ** *     *    *
```
